# Interactions of Sucrose and Trehalose with Lysozyme in Different Media: A Perspective from Atomistic Molecular Dynamics Simulations

**DOI:** 10.1021/acs.molpharmaceut.4c01435

**Published:** 2025-04-25

**Authors:** Inna Ermilova, Jan Swenson

**Affiliations:** Department of Physics, 5264 Chalmers University of Technology, 412 96 Gothenburg, Sweden

**Keywords:** lysozyme, sucrose, trehalose, molecular dynamics, general Amber force field, CHARMM36 force field

## Abstract

Disaccharides are promising additives for stabilizing proteins in, e.g., pharmaceuticals and cryopreserved biomaterials. However, although many studies have shown that disaccharides exhibit such bioprotective and stabilizing properties, the underlying molecular mechanism is still elusive. In this study, we have tried to reach such an understanding by studying lysozyme in aqueous solutions of sucrose or trehalose and various ions (0.1 M Cl^–^, NaCl, and ZnCl_2_) by classical atomistic molecular dynamics (MD). The most important finding for understanding the mechanism of protein stabilization is that the disaccharides, in general, and trehalose, in particular, slow down the protein dynamics by reducing the number of internal hydrogen bonds (both with and without bridging water molecules) in the protein molecules. This reduction of internal protein interactions is caused by disaccharides binding to the protein hydration water, and trehalose forms more hydrogen bonds to water than sucrose. Although it is far from obvious that such a reduction of internal hydrogen bonding in the protein should lead to slower protein dynamics and thereby also a stabilization of the protein, the results show that this is clearly the case. The presence of ions also has some effect on the protein dynamics and stability. Particularly, it is discovered that the ability of sucrose to prevent protein aggregation increases substantially if ZnCl_2_ is added to the solution. The disaccharide and the salt seem to exhibit a synergistic effect in this case. To summarize, we have obtained a molecular understanding of protein stabilization by disaccharides, and why trehalose is more effective than sucrose for this particular system, and the finding is important for understanding how the protein stability in, e.g., pharmaceuticals should be optimized.

## Graphical abstract

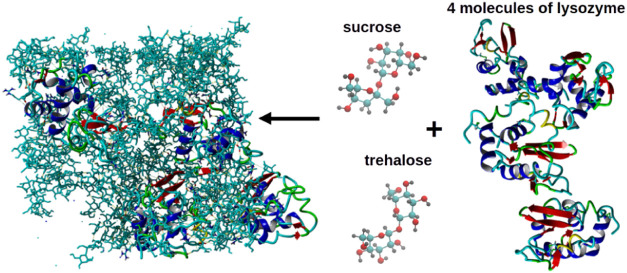

## Introduction

Proteins are known to be important components in biological systems as well as ingredients of various pharmaceutical formulations.^1,2^ Aggregation of these large molecules is undesired. In living organisms, accumulated proteins can cause diseases, while in formulations, such clusters can result in a poor quality of products. An additional property that is not endorsed in industrial applications is the structural instability of these large molecules. Therefore, small molecules that can both inhibit the aggregation and stabilize structures are always of high interest for diverse applications. Such molecules can be disaccharides, lipids, etc.

Disaccharides, in particular, sucrose and trehalose, are already widely used in different formulations in the pharmaceutical,^3^ food,^4^ and cosmetic industries. For instance, sucrose is among the components of the famous mRNA vaccines^5^ against COVID-19. Trehalose is the main component in the eye drops Oxyal.^6^ Nevertheless, despite the widespread utilization of disaccharides for every new formulation, it is challenging to select the right one that would keep the product effective, safe, and long-lasting on its shelf.

When making a choice of a certain disaccharide, various factors are taken into account. The high cryoprotective effect is an important factor when using disaccharides in different formulations.^7^ Storage and freeze-drying at very low temperatures of various pharmaceutical and cosmetic products can result in ice formation, which can negatively affect the texture and other properties of those products.^8–10^

Another such factor is the ability of a disaccharide to preserve the structure.^11^ It can be a structure of a large biomolecule, but it can also be an object containing many different types of molecules, like a lipid nanoparticle (LNP) or a biomembrane.^12–14^

The third desirable function of a disaccharide is its ability to prevent the aggregation of molecules or groups of molecules (e.g., LNPs in some solution). Keeping molecules or LNPs separated from each other can be a key to the efficacy of a certain formulation.^15–17^

In this work, sucrose and trehalose together with an egg protein lysozyme are considered in a computational study by all-atom molecular dynamics (MD) simulations.

Lysozyme is selected as a popular model protein that has been well-studied by various experimental techniques. It is a globular protein^18^ that can be found in, e.g., tears, saliva, and milk.^19^ In humans, lysozyme is a vital part of the immune system as it damages and kills bacteria through multiple mechanisms, such as hydrolyzing specific residues in the peptidoglycan of bacterial cell walls.^20^ The hen egg lysozyme is similar to the human lysozyme, but while the human variant consists of 130 amino acids, the hen egg type has 129 amino acids.^21^ The polypeptide chain is structurally stabilized by four disulfide bonds^22,23^ and includes two domains: one dominated by β-sheets and another composed of mostly α-helices.^23–25^ Located between the two domains is the active site.^25,26^ The structure of the hen egg lysozyme 7VGO^27^ is visualized in Figure 1.

**Figure 1:**
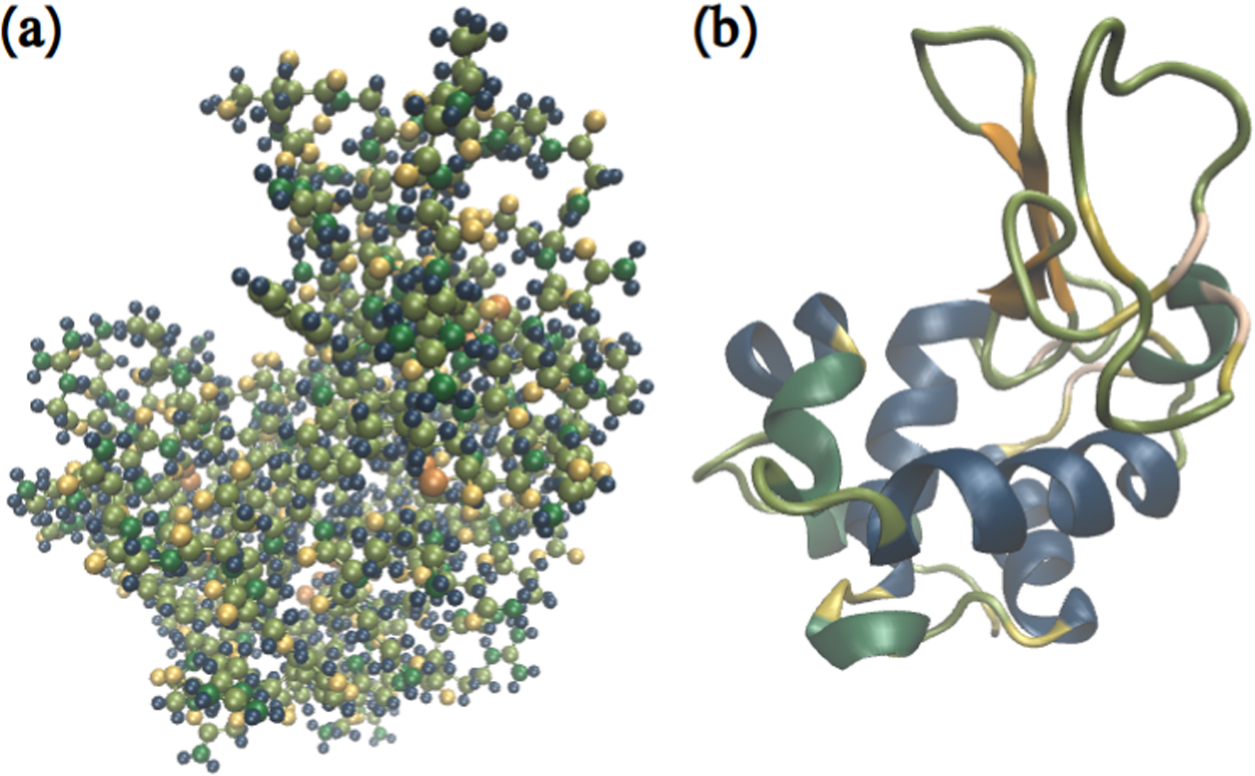
Lysozyme in various representations. (a) Atomistic. (b) Ribbons with secondary structures.

This protein has always been very attractive for experimental studies for various reasons. Considering its properties, perhaps, the reversibility of its denaturation^28^ in a liquid state is of most interest to biophysicists and biochemists. Lysozyme’s therapeutic properties are also intriguing due to its anticancer^29^ and antiviral^30^ activities. For laboratory scientists, this protein is one of the cheapest and most available models.^31–33^ Therefore, there has been extensive research in experimental biophysical chemistry regarding lysozyme.^28,34–39^

Results from experimental works have various suggestions on which disaccharide to select for formulations, but no precise mechanisms of interactions were disclosed.^39^ Knowledge of these mechanisms would help to optimize future formulations.

Therefore, advanced molecular simulations are the right tools for providing such help in the comprehension of the behaviors of pharmaceutical proteins in mixtures with disaccharides. Results from modeling can also be utilized for the explanation of the potential choice of either sucrose or trehalose for a selected ratio of compounds.

Lysozymes in mixtures with disaccharides (with sucrose, trehalose, and maltose) were studied earlier by Lerbret et al.,^40^ but each mixture contained only one protein molecule, which gives no information about possible interactions between proteins. In their results, it was revealed that trehalose was more excluded from the protein surface than maltose and sucrose.

Trehalose–protein interactions where lysozyme was used as a model were the topic of work by Lins et al.^41^ 0.5 M concentration of the disaccharide was utilized in the presence of a single protein. They concluded that trehalose was not a complete dehydrating lysozyme but was acting as a coating for it.

Fedorov et al.^42^ investigated aqueous mixtures of lysozyme and trehalose. Their simulations were also designed for single proteins. It was discovered that trehalose binds to the surface of lysozyme, but again, there was no information about inter-protein interactions.

Simončič et al.^43^ also simulated single lysozyme molecules but with sucralose and sucrose. They found that sucralose was a better preservative for protein than sucrose.

In contrast to previous works, in this project, larger systems are considered for modeling. Atomistic MD simulations are performed for 4 proteins in each mixture. This can help to understand whether disaccharides can inhibit the aggregation of lysozyme. Counterions of Cl^–^ and salts (NaCl, ZnCl_2_) are used for finding out how they can affect the protein dynamics and the different interactions between the protein, disaccharide, and water.

## Methods and Models

### All-Atom MD Simulations

Starting configurations were designed in the following way. In cubic boxes with a side of 25 nm, 4 proteins were randomly placed, keeping a distance of around 1 nm between them in order to avoid initial aggregation due to too close locations. After that, disaccharides were added in boxes containing only 4 proteins. The model for the protein was taken from the CHARMM36 ^44^ force field, while models for disaccharides were taken from the work by Ahlgren et al.^45^

After the addition of disaccharides, water of TIP 3p^46,47^ and ions were added, where the amount of ions was equal to the concentration of 0.1 M NaCl (which is not valid for counterions of Cl^–^ used for neutralizing the total charge of the system). The idea was to design systems corresponding to the following mass ratios:

- *m*(Lysozyme)/*m*(Water) = 1:2.7;
- *m*(Lysozyme)/*m*(Sucrose/Trehalose)/*m*(Water) = 1:1.3:2.7.

The final compositions of the systems are presented in Table 1.

**Table 1:** Compositions of Simulated Systems Using Atomistic Models^a^

system	number of disaccharides	number of positive ions	number of negative ions	number of molecules
LYS	0	0	32	8596
LYS + SUC	223	0	32	8478
LYS + TRE	223	0	32	8478
LYS + NaCl	0	35	67	8596
LYS + SUC + NaCl	223	34	66	8478
LYS + TRE + NaCl	223	34	66	8478
LYS + ZnCl_2_	0	18	68	8596
LYS + SUC + ZnCl_2_	223	17	66	8478
LYS + TRE + ZnCl_2_	223	17	66	8478

a For simplifying the discussion, systems will be labeled with abbreviations: LYS—lysozyme, SUC—sucrose, TRE—trehalose.

Before running actual production runs, every system was equilibrated using GROMACS-2019 ^48^ as an MD engine. Production runs were performed in the NPT^49^ ensemble using the isotropic pressure coupling scheme for 200 ns with a time-step of 2 fs and the integrator leapfrog.^50^ Berendsen^51^ barostat was utilized to maintain a pressure of 1.013 bar with a coupling constant of 10 ps and a compressibility of 4.5 × 10^–5^ bar^–1^. The temperature was kept at 310.15 K by a velocity rescale^52^ thermostat with a coupling constant of 0.5 ps. A cutoff distance of 1.2 nm was employed for Coulumb, Lennard-Jones, and short-range neighbor interactions with the cutoff scheme Verlet.^53^ The trajectory was recorded every 4 ps. LINCS^54^ algorithm with 12 iterations was utilized for constraining bonds. Long-range electrostatics was handled by the particle mesh Ewald^55^ algorithm.

The last frames from equilibrations were used as starting configurations for production runs with the NVT^56^ ensemble using the velocity rescale^52^ thermostat, which helps to maintain the exact canonical sampling. Every simulation was 500 ns long, and the output for the trajectory was made every 2 ps. All other settings, including the thermostat and relevant constant, were the same as during the equilibration, except that a barostat was not used.

## Statistical Software and Calculations

For performing analysis of simulated data, statistical tools from the programming language Python-3 were used. In particular, routines from packages NumPy, SciPy, and matplotlib were utilized for statistical calculations and plotting graphs.^57,58^

For calculations of correlations, Pearson’s and Spearman’s correlation coefficients were used.

Pearson’s correlation^59,60^ coefficient is used to measure the linear correlation between two data sets. Where absolute values between 0.5 and 1 suggest strong correlation, values in the range of 0.3 and 0.49 indicate moderate correlation, weak correlation can be concluded from coefficients in the range below 0.29, and no correlation can be deduced from values close to 0.

Spearman’s correlation^59,61^ coefficient is applied for finding out the monotonicity of the relationship between two data sets. The classification is similar to the one used for Pearson’s correlation coefficient.

For each correlation coefficient, its *P*-value is utilized, which is the value of the probability that the computed correlation is coming from an uncorrelated data set, meaning that the coefficient is false.

## Results

### All-Atom MD Simulations

Before discussing statistical results, it is important to have a look at snapshots from the last frames of the systems. Figure 2 demonstrates snapshots for all systems. Regardless of what ions were present in each system, lysozyme appears to have a larger tendency to aggregate in the absence of disaccharide (Figure 2a,d,g). The disaccharides separate the protein molecules, but there are no significant visual differences in snapshots between the systems containing sucrose or trehalose.

**Figure 2:**
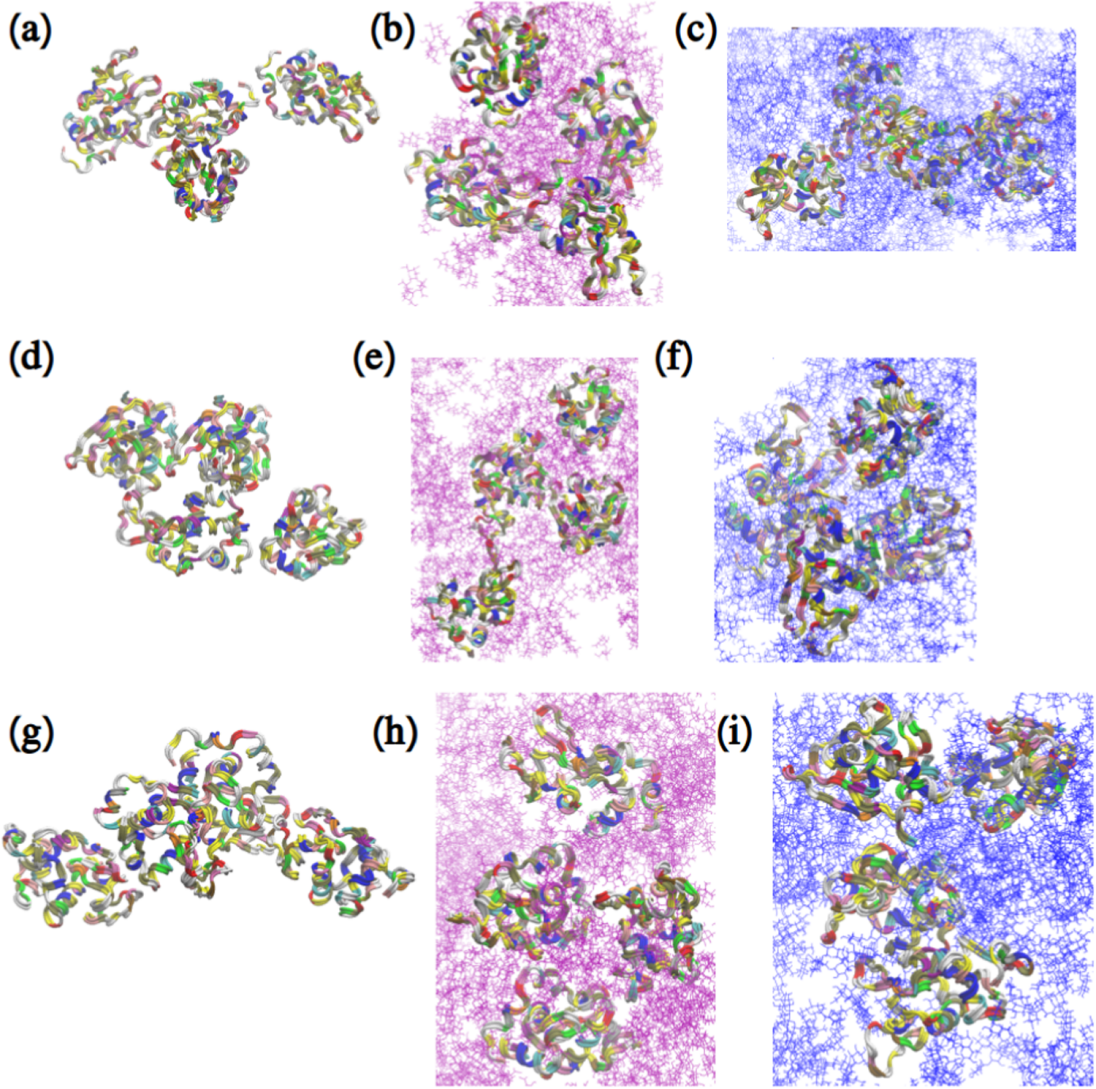
Snapshots from atomistic MD simulations of the following systems: (a) LYS. (b) LYS + SUC. (c) LYS + TRE. (d) LYS + NaCl. (e) LYS + SUC + NaCl. (f) LYS + TRE + NaCl. (g) LYS + ZnCl_2_. (h) LYS + SUC + ZnCl_2_. (i) LYS + TRE + ZnCl_2_. Water and ions have been omitted for clarity. Proteins are presented as ribbons. Sucrose and trehalose are presented as thin purple and blue molecules, respectively.

#### Root-Mean-Square Deviation (RMSD) and Radius of Gyration

RMSD tells us about how similar two protein structures are by comparing their superimposed atomic coordinates. High values of RMSD indicate large changes in the structure compared to the reference structure. The statistics over time also provide information about the dynamics of lysozyme.

In order to find out how stable proteins are in each mixture, RMSDs for every single chain of lysozyme were computed and are presented in Figures S1–S3 of the Supporting Information (ESI). The average RMSD profiles for all proteins in each system are listed in Figure 3. Lysozyme in aqueous solutions without disaccharides appears to be the most unstable due to the highest values of RMSD. For systems with only counter-chloride ions, there is basically no difference in the RMSD of lysozyme between the two disaccharide-containing systems. The presence of NaCl makes trehalose a better preservative due to smaller values of RMSD compared to sucrose. In solutions with ZnCl_2_, the presence of disaccharides does not seem to play a big role because RMSDs for all 3 systems appear to be similar. Nevertheless, all observed fluctuations of RMSD were not dramatic, as values were oscillating in the range of 0.1–0.2 nm.

**Figure 3:**
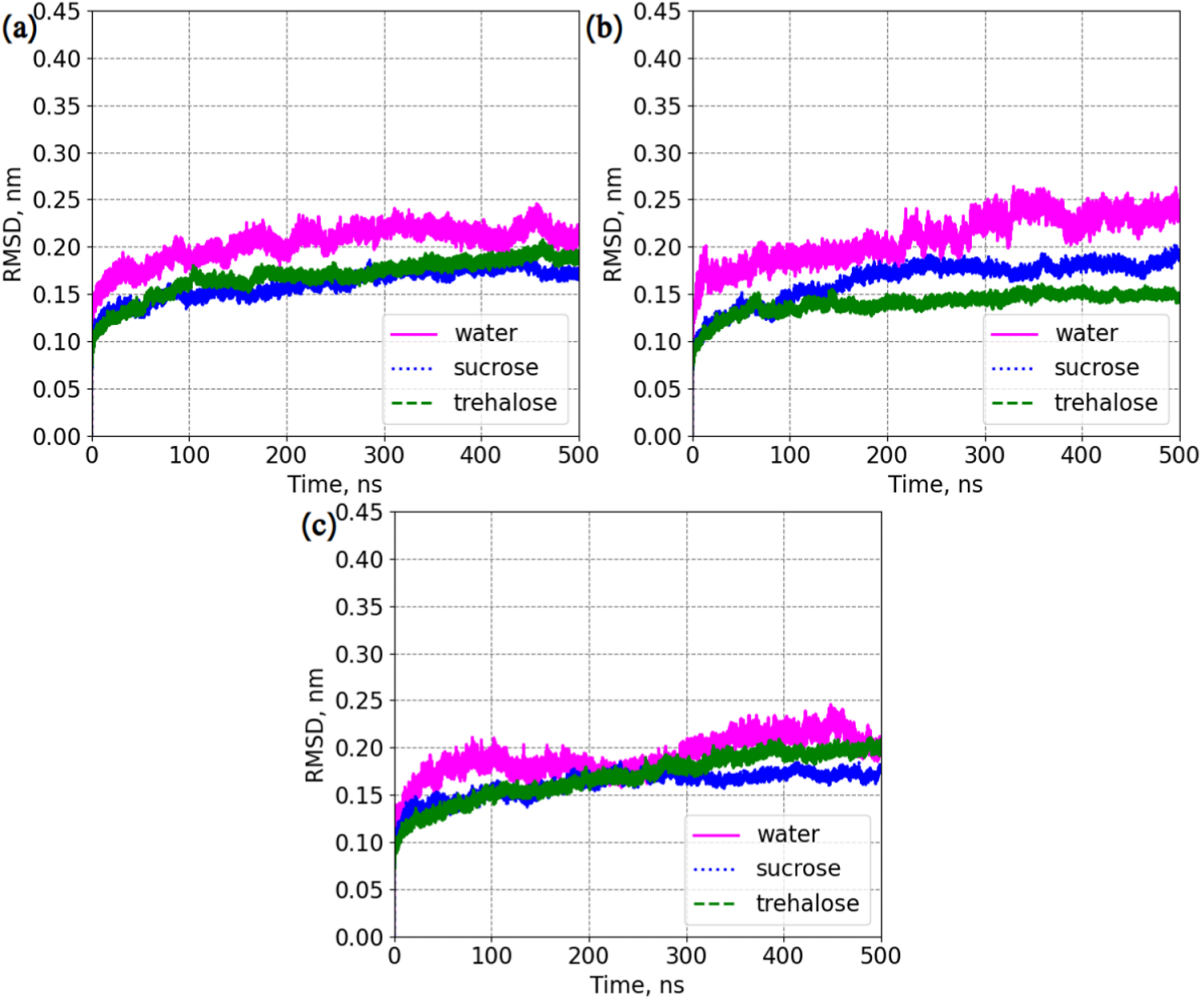
Average RMSD for lysozyme. (a) Systems: LYS, LYS + SUC, and LYS + TRE. (b) Systems: LYS + NaCl, LYS + SUC + NaCl, and LYS + TRE + NaCl. (c) Systems: LYS + ZnCl_2_, LYS + SUC + ZnCl_2_, and LYS + TRE + ZnCl_2_. “Water” stands for systems without disaccharides. RMSD was computed for each protein molecule and then averaged over the 4 molecules.

The evolution of the radius of gyration was also computed for the protein (Figures S4–S7 in ESI). In all simulated systems, differences in values were rather negligible.

### Self-Intermediate Scattering Functions (SISF) and Relaxation Times

Self-intermediate scattering functions (SISF) give additional information about the mobilities of the molecules in the system. They are calculated from the self-part of Van Hove’s function.^62–64^ For a protein, the most essential part responsible for its secondary structure is the backbone. Therefore, to determine whether disaccharides can stabilize protein molecules, it is enough to calculate backbone relaxation times from SISFs for some selected values of the scattering vector *q*.

Figure 4 demonstrates relaxation times for 4 selected *q*-values for the backbone of the protein. The values were obtained by fitting a single exponential function to each SISF (as was done by Gilbert et al.^65^), as presented in Figures S8–S11. Lysozyme has the fastest dynamics in aqueous solutions without disaccharides, while in the presence of trehalose, it has the slowest dynamics.

**Figure 4:**
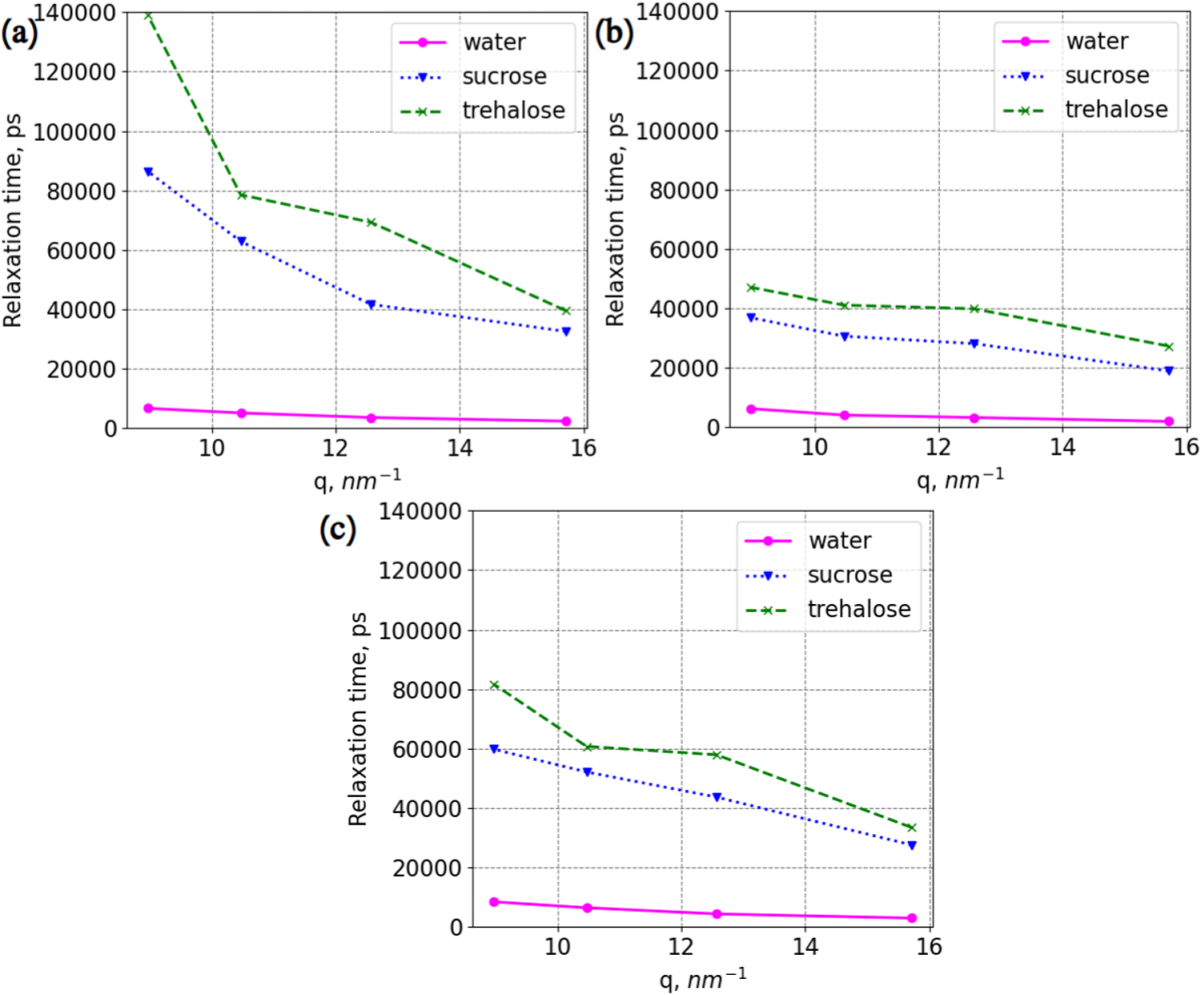
Relaxation times for the backbone of lysozyme. (a) Systems: LYS, LYS + SUC, and LYS + TRE. (b) Systems: LYS + NaCl, LYS + SUC + NaCl, and LYS + TRE + NaCl. (c) Systems: LYS + ZnCl_2_, LYS + SUC + ZnCl_2_, and LYS + TRE + ZnCl_2_. “Water” stands for systems without disaccharides. Error bars are within the markers.

The presence of ions also affects the motion of lysozyme. In solutions without disaccharides, the protein’s backbone has the slowest dynamics in the presence of ZnCl_2_, and it relaxes faster with NaCl. With disaccharides, the protein’s backbone has the slowest dynamics when only Cl counterions are added, and it has the shortest relaxation times with NaCl.

#### Hydrogen Bonds, Total Number of Contacts, and Radial Distribution Functions (RDFs)

Hydrogen bonding plays a key role in the behavior of the simulated systems. The protein structure depends on hydrogen bonds between amino-acid residues. Figure 5 shows hydrogen bonds between different molecules in the modeled mixtures.

**Figure 5:**
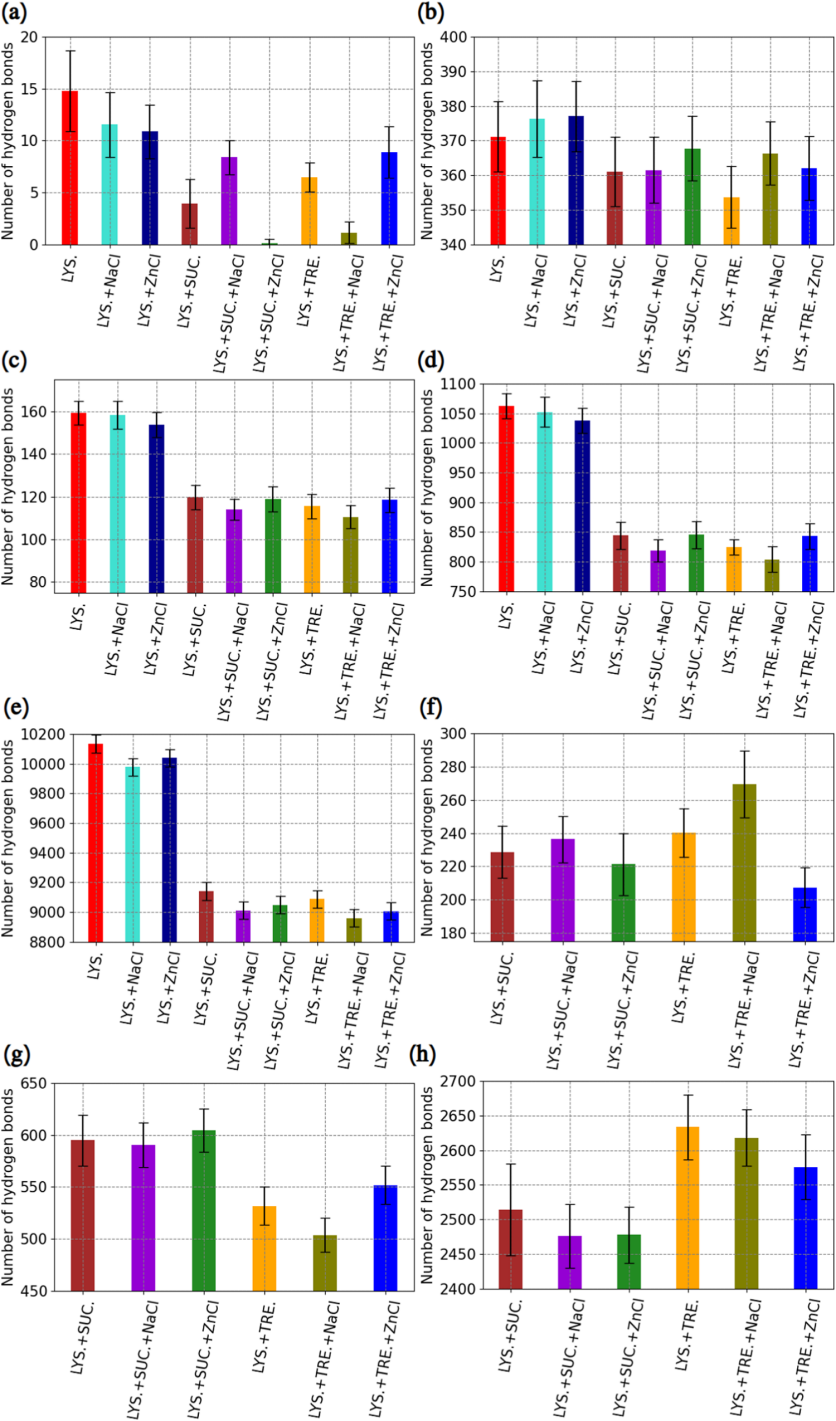
Average number of hydrogen bonds in the systems. (a) Protein–protein: inter-molecular. (b) Protein–protein: intra-molecular. (c) Protein–water–protein (here, values are per 1 protein molecule): bridging intra-protein bonds with water. (d) Protein–water. (e) Water–water. (f) Protein–disaccharide. (g) Disaccharide–disaccharide. (h) Disaccharide–water. Systems’ names are on the *x*-axis, named after Table 1. Error bars represent the standard deviation. The standard error was 0.02% for every simulation. In the figure legend on the *x*-axis, “ZnCl” is used as an abbreviation for ZnCl_2_. Average numbers of protein–water and water–water hydrogen bonds per water molecule, corresponding to parts (c), (d), and (e), are presented in Figure S12 of the SI.

When considering protein–protein hydrogen bonds, it is important to distinguish between inter- and intra-molecular ones. The number of inter-molecular hydrogen bonds (Figure 5a) tells us about the affinity between different lysozyme molecules (each simulation had 4 of them). In systems without disaccharides, these numbers are the highest because there is no hindrance to potential binding. This finding is coherent with results reported by Valente et al.,^66^ where by self-interaction chromatography experiments, it was discovered that all disaccharides reduced protein inter-molecular attraction.

Moreover, from our data in the presence of sucrose and ZnCl_2_, the protein molecules are almost perfectly separated, as the number of hydrogen bonds is close to 0, while trehalose is less successful in the presence of the same salt. Instead, trehalose inhibits protein aggregation best in the mixture with NaCl, where sucrose is not able to separate different lysozyme molecules. In the presence of only counterions of Cl^–^, both disaccharides perform similarly. Regarding the higher ability of sucrose in the presence of ZnCl_2_ to separate protein molecules, it is important to note that there are less hydrogen bonds on average in an aqueous solution of lysozyme and ZnCl_2_ compared to a pure aqueous solution of lysozyme. This implies that the efficacy of sucrose is a result of the synergistic effects of the disaccharide and the salt. In fact, in a recent experimental work, Rogowska et al.^67^ discovered that Zn^2+^ ions could stabilize hen egg white lysozyme.

Intra-molecular hydrogen bonds have another importance for preserving the protein structure, as their internal network is responsible for holding secondary structures. There are more internal hydrogen bonds within the lysozyme in mixtures without disaccharides (Figure 5b). However, within the large standard deviations, there is no significant difference in the number of hydrogen bonds between the sucrose- and trehalose-containing systems, although the average values for trehalose are smaller than for sucrose in all systems except those with NaCl.

The internal structure of proteins in the presence of water is also held by hydrogen bridging bonds or so-called bifurcated^68–70^ hydrogen bonds. They are formed between parts of lysozyme, where water with ions plays the role of connecting atoms. The only way to isolate such hydrogen bonds is by creating new index files with the following groups: for each protein molecule, a separate group is made (4 groups), and then, a group with each protein molecule including water and ions is created (4 groups). The resulting values of protein–water–protein (this name is chosen for intra-molecular bonds of a protein, which occur through atoms of water molecules, acting as connecting units for the internal structure of lysozyme) bridging hydrogen bonds are computed using the following eq 1:

1 $$HB_{\mathrm{intra},p-w-p,\mathrm{bridging}}=\frac{1}{4}\left(\sum_{i=1}^{4}HB_{\mathrm{intra},p+w,i}+\sum_{i=1}^{4}HB_{\mathrm{intra},p,i}\right)-HB_{w-w}$$

Here, “intra” stands for intra-protein, “p”—protein, “w”—water and ions, and “HB”—number of hydrogen bonds. Since the resulting value is divided by the number of lysozyme molecules (which equals 4) before the number of water–water hydrogen bonds is subtracted, it gives us the average number of bridging hydrogen bonds per protein molecule. Computed values are shown in Figure 5c. From the figure, it follows that in systems without disaccharides, there are more bridging hydrogen bonds in the internal structure of the protein. From the average values, it can be concluded that in mixtures of trehalose, the number of such hydrogen bonds is slightly smaller than in the presence of sucrose, although the differences are within the standard deviations. The addition of ZnCl_2_ results in a decrease of the number of bridging hydrogen bonds in the system without disaccharides, and the highest number of such bonds is obtained for the sample with only counterions of Cl^–^. However, also in this case, the differences are sufficiently small to be within the standard deviations.

Similar calculations were performed by replacing water and ions with disaccharides in order to investigate whether they are forming bridging bonds within the protein. However, that was not the case, probably due to their larger size compared to water molecules.

Considering further interactions of lysozyme with water and ions, from Figure 5d, it can be concluded that disaccharides decrease the number of protein–water hydrogen bonds compared to the corresponding systems without them. This can be explained by the binding of sucrose and trehalose to lysozyme molecules (Figure 5f), where from the average numbers of hydrogen bonds, it can be concluded that ZnCl_2_ inhibits associations between disaccharides and protein, while NaCl promotes it.

As water and ions affect the behavior of proteins and disaccharides, the latter can also influence the interactions between water and ions. Figure 5e presents hydrogen bonds between water molecules in each system. The highest number of hydrogen bonds is obtained for mixtures without disaccharides, where the highest average value is for the system with only counterions of Cl^–^. In mixtures without disaccharides, the lowest average number of water–water hydrogen bonds is observed for the system with NaCl. A comparison of systems with either sucrose or trehalose shows that in the presence of trehalose, the average number of hydrogen bonds between water molecules was smaller than in mixtures with sucrose. The smallest average value of water–water hydrogen bonds is found for lysozyme with trehalose and NaCl, while the highest one is observed for lysozyme with sucrose and counterions of Cl^–^.

From differences between the numbers of protein–water (Figure 5d) and protein–disaccharide (Figure 5f) hydrogen bonds, it can be concluded that there still is a preferential hydration of the protein. Moreover, trehalose and sucrose have a tendency to bind water themselves, where sucrose binds less water than trehalose (Figure 5h).

The disaccharides also form some hydrogen bonds with each other (Figure 5g), where there are more sucrose–sucrose hydrogen bonds than there are trehalose–trehalose. This is coherent with earlier findings by Ermilova et al.^71^

The number of contacts between amino-acid residues is another characteristic that can explain possible mechanisms of interactions in the studied systems. Figure 6 and Table 2 demonstrate the average numbers of contacts per amino-acid residue. The figure shows that there are more contact points within the protein molecules in mixtures without disaccharides, although Table 2 shows that there are no dramatic differences in the numbers of contacts among the systems if the standard deviations are taken into account.

**Figure 6:**
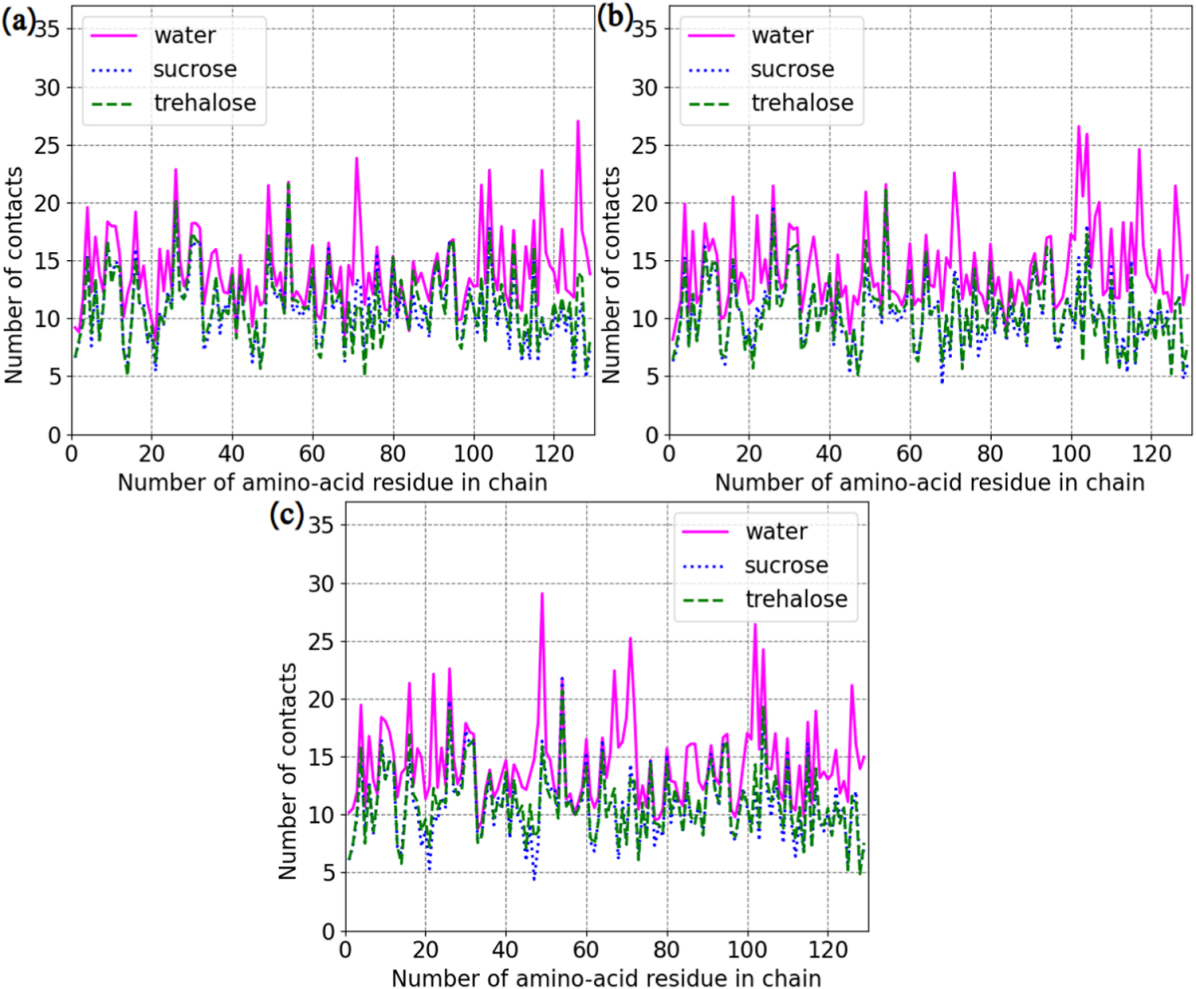
Average number of contacts per amino-acid residue per protein molecule. (a) Systems: LYS, LYS + SUC, and LYS + TRE. (b) Systems: LYS + NaCl, LYS + SUC + NaCl, and LYS + TRE + NaCl. (c) Systems: LYS + ZnCl_2_, LYS + SUC + ZnCl_2_, and LYS + TRE + ZnCl_2_. “Water” stands for systems without disaccharides.

**Table 2:** Average Number of Contacts per Amino-Acid Residue

system	average number of contacts per amino-acid residue
LYS	14.00 ± 3.45
LYS + SUC	10.87 ± 3.13
LYS + TRE	11.07 ± 3.14
LYS + NaCl	14.22 ± 3.54
LYS + SUC + NaCl	10.46 ± 3.15
LYS + TRE + NaCl	10.54 ± 3.12
LYS + ZnCl_2_	14.39 ± 3.67
LYS + SUC + ZnCl_2_	10.79 ± 3.22
LYS + TRE + ZnCl_2_	11.01 ± 2.99

Additional information about the interactions between protein molecules and disaccharides can be obtained from RDFs (which is also *g*(*r*)) between the centers of mass of disaccharides and amino-acid residues.

Figure 7 demonstrates such RDFs for every studied mixture with sucrose and trehalose. Despite similar appearances of patterns, there are differences. In mixtures without salts, trehalose and sucrose associate with roughly 54 amino-acid residues. When NaCl is present, the number of associated amino-acid residues with sucrose is 66, while for trehalose, it is 57. In ZnCl_2_, sucrose can possibly bind to 52 residues, while this number for trehalose is 47. Thus, the overall sucrose binds slightly more amino-acid residues.

**Figure 7:**
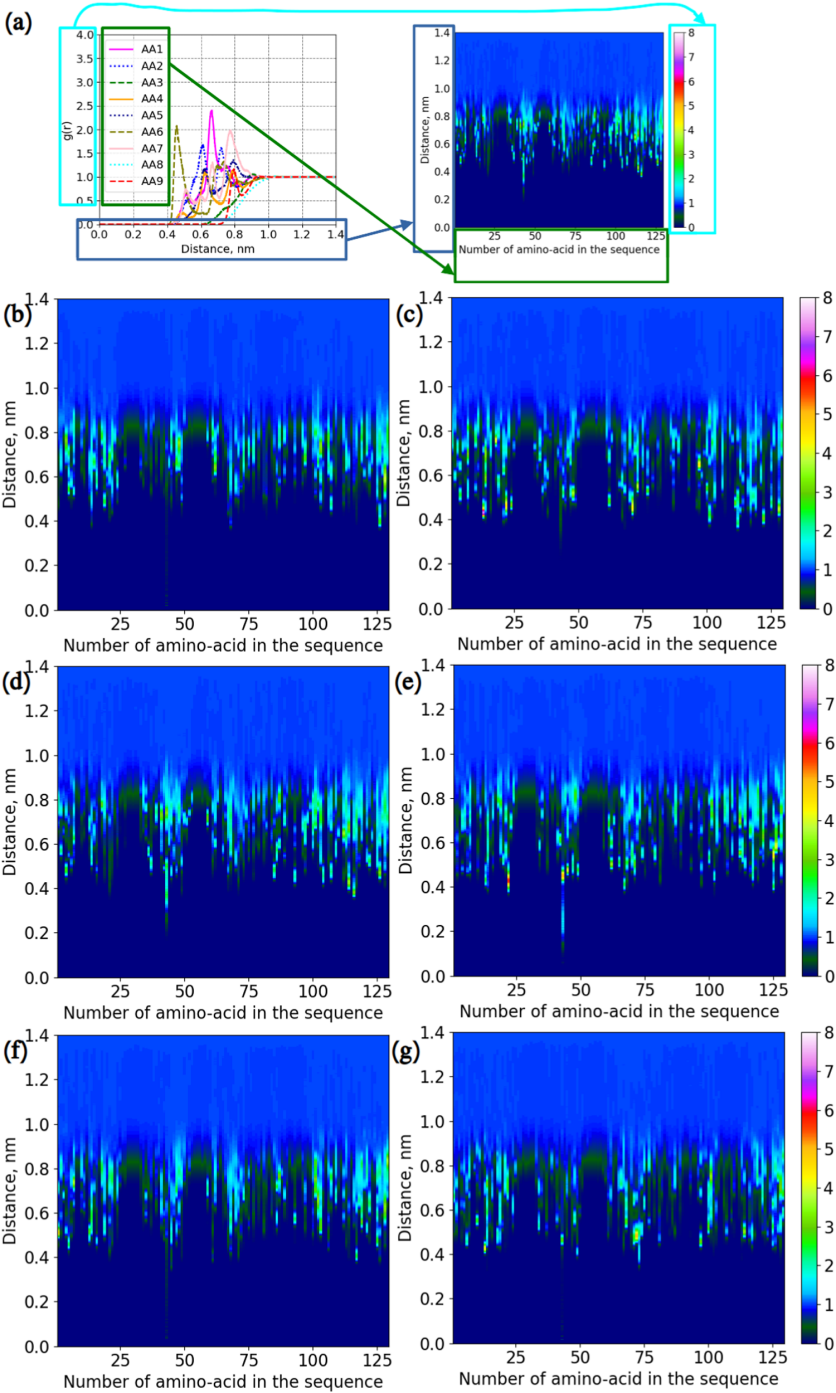
RDFs for protein–disaccharide interactions computed between the centers of mass of amino-acid residues and disaccharides. (a) Illustration of how colormaps were created. (b) System LYS + SUC. (c) System LYS + TRE. (d) System LYS + SUC + NaCl. (e) System LYS + TRE + NaCl. (f) System LYS + SUC + ZnCl_2_. (g) System LYS + TRE + ZnCl_2_.

## Discussion

The presented data indicate that trehalose is a better agent for slowing down the dynamics of systems with lysozyme. In order to understand why this happens and detect potential mechanisms, one needs to investigate possible correlations in the presented data to correlate the dynamic stability of the protein with structural characteristics.

For providing proper statistical determinations for this discussion, well-known Pearson’s and Spearman’s correlations were utilized. Figure 8 demonstrates significant correlations obtained from the presented data.

**Figure 8:**
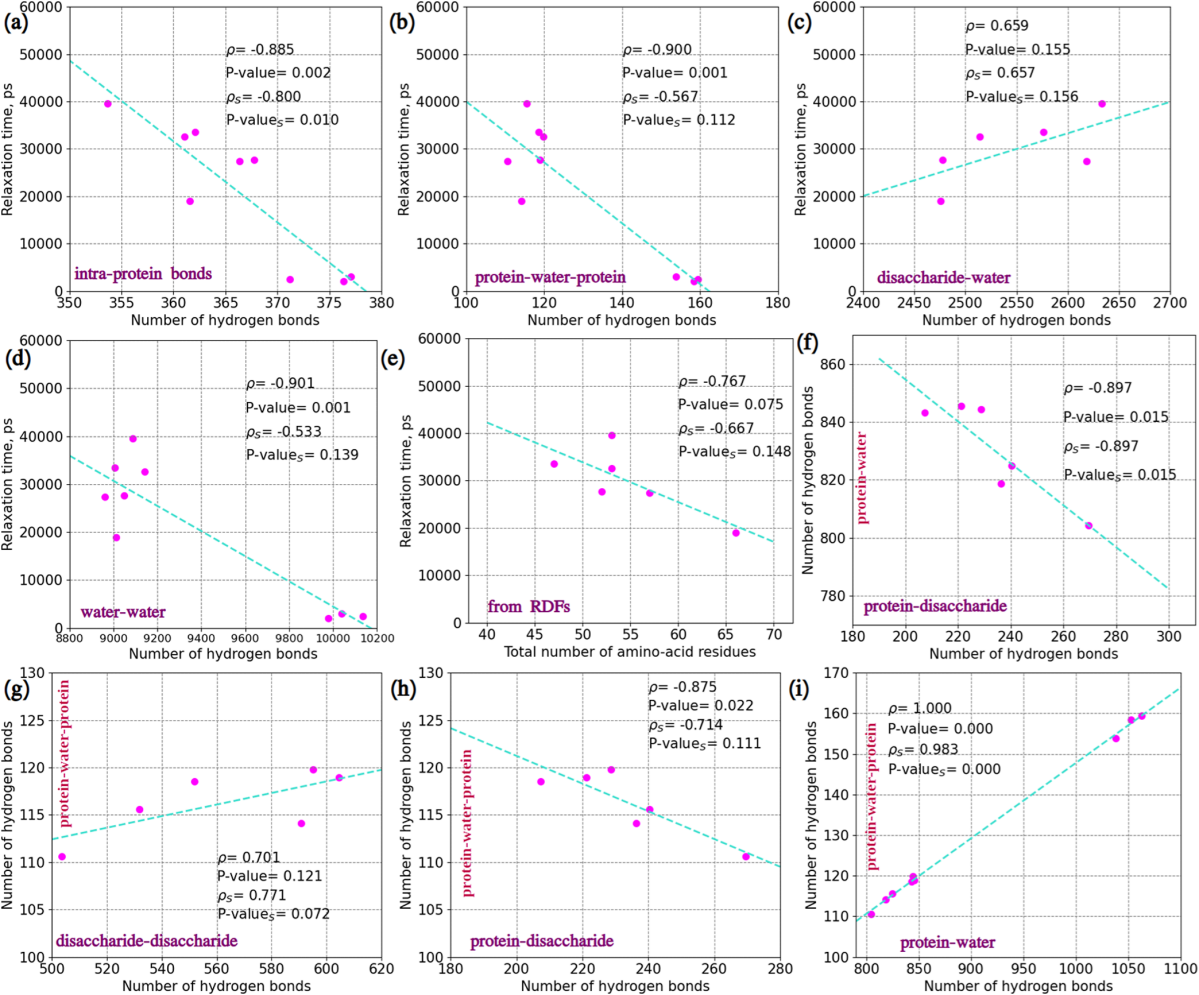
Correlations. (a) Relaxation times and number of intra-protein hydrogen bonds. (b) Relaxation times and number of protein–water–protein bridging hydrogen bonds. (c) Relaxation times and number of disaccharide–water hydrogen bonds. (d) Relaxation times and number of water–water hydrogen bonds. (e) Relaxation times and the total number of amino-acid residues associated (data from RDFs) with disaccharides. (f) Protein–disaccharide and protein–water hydrogen bonds. (g) Disaccharide–disaccharide and protein–water–protein bridging hydrogen bonds. (h) Protein–disaccharide and protein–water–protein bridging hydrogen bonds. (i) Protein–water and protein–water–protein bridging hydrogen bonds. Relaxation times were computed for *q* = 15.71 nm^–1^. Here, ρ is the Pearson’s correlation coefficient, and *P*-value is the probability of the correlation being wrong. ρ_S_ is the Spearman’s correlation coefficient, and *P*-value_S_ is the probability of the correlation being wrong. Pink points represent the data points; turquoise lines are fitting lines.

From Figure 8a,b and the high absolute values of Pearson’s and Spearman’s correlation coefficients together with their low *P*-values, it follows that the protein relaxation time is strongly dependent on internal bonds in the protein molecules, including the bridging bonds where water and ions can act as possible bridges^72,73^ between atoms of lysozyme: the dynamics of the protein backbone is slower when there are less intra-molecular and internal bridging hydrogen bonds. Thus, the disaccharide molecules are not only disrupting the protein hydration shell and slowing down its dynamics but also reducing the number of internal hydrogen bonds in the protein. This is an important finding for understanding how disaccharides reduce the protein dynamics and stabilize proteins.

Another important relation to the protein relaxation time is the strong positive correlation with disaccharide–water hydrogen bonds presented in Figure 8c: a higher number of those bonds is related to a longer relaxation time. Consequently, this describes the potential ability to dehydrate the internal protein structure and contribute to the result shown in Figure 8b. However, recalling conclusions from the work by Lins et al.^41^ and considering the numbers of protein–water hydrogen bonds, it can also be deduced that the disaccharides act as a coating around the lysozyme rather than as a dehydrating agent, which can also be a reason behind the slower relaxation of the internal protein structure.

It is known that the dynamics of water have an effect on other components of biological systems. There are more hydrogen bonds in more diluted systems, which leads to faster protein dynamics. Figure 8d exhibits the strong negative Pearson’s and Spearman’s correlations between the number of water–water hydrogen bonds and the relaxation time, which is fully consistent with the literature.

The last strong negative correlation with the relaxation time is with the number of associated amino-acid residues obtained from RDFs: the disaccharide with the highest ability to inhibit the dynamics of lysozyme’s backbone is the one which binds to fewer residues, which is trehalose (Figure 8e).

In order to disclose more details on the mechanisms of protein stabilization, correlations between hydrogen bonds were calculated as well. Figure 8f shows a strong negative correlation between protein–disaccharide and protein–water hydrogen bonds: there are less protein–water hydrogen bonds in systems with more protein–disaccharide hydrogen bonds, which is a natural consequence of the fact that disaccharide molecules replace water molecules at the protein surface.

Protein–water–protein bridging (or bifurcated) hydrogen bonds are in strong positive and strong negative correlations with disaccharide–disaccharide and protein–disaccharide hydrogen bonds, respectively (Figure 8g,h). This is also a consequence of that hydroxyl groups of the disaccharides are replacing water molecules in the protein hydration shell without becoming bridging units themselves, which thereby decreases the number of those bifurcated hydrogen bonds. This is also confirmed by a strong positive correlation between the bridging hydrogen bonds and the protein–water hydrogen bonds (Figure 8i).

Finalizing this discussion, mechanisms of lysozyme preservation can be proposed for the studied mass ratios of protein, disaccharides, water, and ions. In the simulated systems, a well-preserved lysozyme is the one with the smallest number of intra-molecular hydrogen bonds. Thus, the stabilized backbone is less hydrated compared to less stable structures, where more bridging (bifurcated) hydrogen bonds with water can be observed. This implies that hydroxyl groups of the disaccharides replace and bind water from the hydration shell of the protein, which results in suppressed dynamics of the shell, as disaccharides both have slower dynamics than water, as well as slowing down the hydration water,^74^ which in turn slows down the protein motions.^75^ Therefore, sucrose/trehalose together with water molecules form protective layers around the protein molecules, which ensures protein preservation.

Trehalose has properties that are better at stabilizing the lysozyme structure than sucrose at the studied protein-sugar mass ratios. On the other hand, if there is a strong demand to use sucrose as a preservative for lysozyme, then it is valuable to consider its combination with zinc ions, which have the ability to improve the performance of sucrose.

The charge of zinc ions is twice as high as the charge of sodium ions, which results in their ability to bind more strongly to negatively charged parts of lysozyme, preventing sucrose from binding to the protein. Zinc is also playing a significant role in preventing the aggregation of insulin^76^ and inhibiting amyloid fibril formation of hen egg lysozyme.^77^ The perturbed homeostasis of this metal is known to have implications for age-related diseases.^78^ Since the biological inorganic chemistry of zinc ions is still not well-understood,^79^ it is valuable to invest more effort into research on possibilities to utilize these ions for protein stabilization.

## Conclusions

MD simulations on an atomistic level were performed in this work for aqueous solutions of lysozyme with disaccharides or various salts. The results show that trehalose slows down the protein dynamics more than sucrose, and the reason for this seems to be that trehalose forms more hydrogen bonds to water and thereby disrupts the protein hydration of water more than sucrose. Sucrose binds to more amino-acid residues than trehalose, but it cannot form hydrogen bonds to the same amount of water as trehalose due to the fact that sucrose has a stronger affinity to itself, as shown earlier by Ermilova et al.^71^ The stronger hydrogen bonding of trehalose to water seems, in turn, to result in a reduced number of internal protein hydrogen bonds, both with and without bridging water molecules. Thus, somewhat surprisingly, there is a strong correlation between an increasing protein relaxation time and a decreasing number of internal protein hydrogen bonds. This is an important finding for understanding the rules of disaccharides, in general, and trehalose, in particular, for protein stabilization. The addition of the disaccharides to the protein solution seems to have an effect on the protein dynamics similar to that of a dehydration of the protein without causing the detrimental effects of dehydration.

Effects of the presence of salts or counterions on protein dynamics were also disclosed. The slowest relaxation of lysozyme is observed when salts are absent and only counterions of Cl^–^ are added to the mixtures, while the fastest motion of the protein is seen in the presence of NaCl. Moreover, ions affect the ability of disaccharides to separate different protein molecules and, thereby, prevent protein aggregation. In mixtures with only Cl^–^ counterions and with ZnCl_2_, sucrose prevents the aggregation of lysozyme better than trehalose. In fact, Zn^2+^ ions are known as inhibitors of the amyloid fibril formation of hen egg white lysozyme.^77^ A combination of these ions and lysozyme has also shown antimicrobial activities.^67^ Therefore, it is valuable to consider lysozyme, encapsulated with the help of sucrose and Zn^2+^ ions, for pharmaceutical applications.

In the case of protein solutions with trehalose, protein aggregation is most effectively prevented by adding NaCl. This is also an important finding since NaCl is the most common salt in physiological systems and, furthermore, is used by the computational community to mimic the physiological acidity of the environment.

To summarize, the findings we have obtained in this study are important for understanding how proteins in, e.g., pharmaceuticals should be stabilized for maintaining the desired properties over time.

## Supplementary Material



## Notes

Figures of RMSDs for every protein; figures of radius of gyration for each protein; figures of self-intermediate scattering functions; and average number of hydrogen bonds per 1 water molecule (PDF)
