# Supplementary material for: Interactions of Sucrose and Trehalose with Lysozyme in Different Media: A Perspective from Atomistic Molecular Dynamics Simulations
Source: Mol Pharm. 2025 Apr 25;22(6):2997–3009. doi: 10.1021/acs.molpharmaceut.4c01435 (PMC12135068; doi:10.1021/acs.molpharmaceut.4c01435)
Supplement: Supplementary file 1 [file mp4c01435_si_001.pdf]

Electronic Supplementary Information for "Interactions of sucrose and trehalose with lysozyme in different media: a perspective from atomistic molecular dynamics simulations"

Inna Ermilova, [a,*], Jan Swenson [a]

[a] *Department of Physics, Chalmers University of Technology, 412 96 Gothenburg, Sweden*
[*] *Corresponding author: inna.ermilova@chalmers.se; inna.ermilova@gmail.com*

Contents

1 Root mean square deviations (RMSDs) **S2**

2 Radius of gyration **S3**

3 Self-intermediate scattering functions **S5**

4 Average number of hydrogen bonds per 1 water molecule **S7**

1 Root mean square deviations (RMSDs)

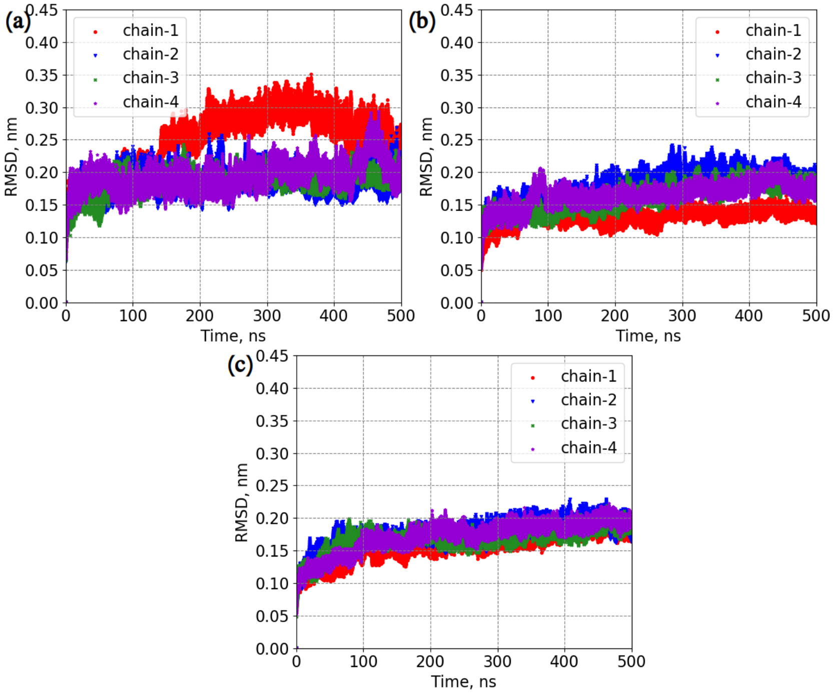

Figure S1 RMSD for protein chains. (a) Systems: LYS. (b) LYS.+SUC. (c) LYS.+TRE. RMSD was computed for each protein.

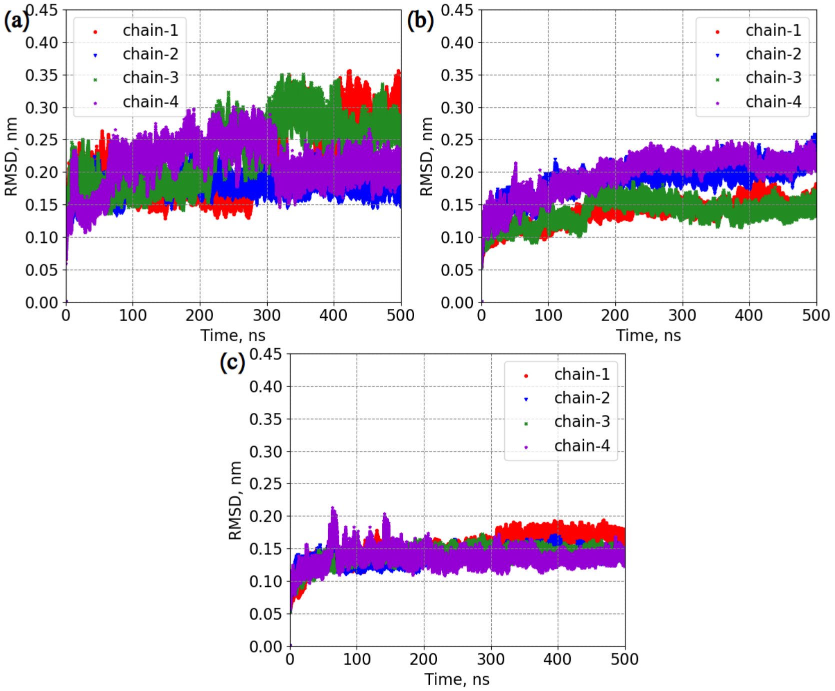

Figure S2 RMSD for protein chains. (a) Systems: LYS.+NaCl. (b) LYS.+SUC.+NaCl. (c) LYS.+TRE.+NaCl. RMSD was computed for each protein.

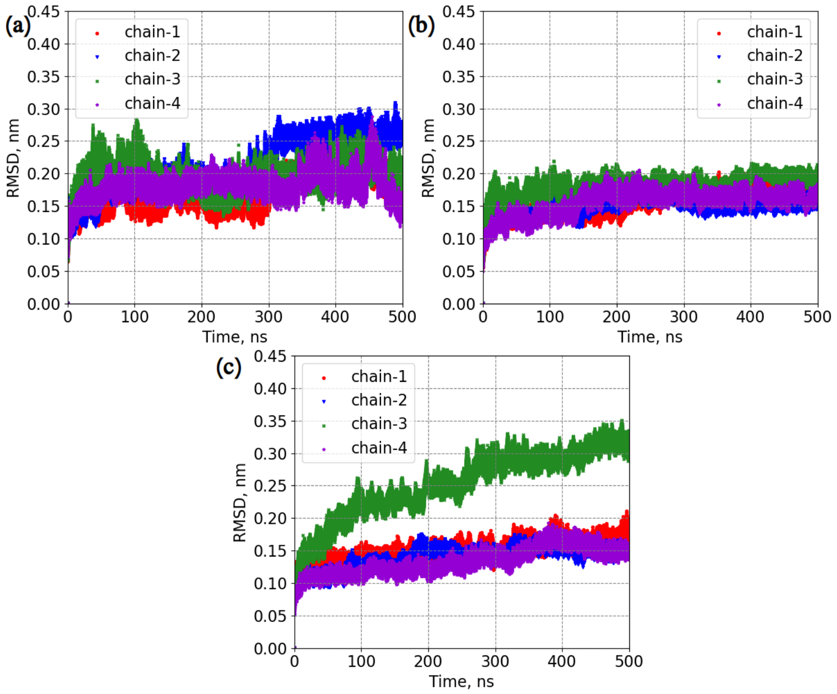

Figure S3 RMSD for protein chains. (a) Systems: LYS.+$ZnCl_2$. (b) LYS.+SUC.+$ZnCl_2$. (c) LYS.+TRE.+$ZnCl_2$. RMSD was computed for each protein.

2 Radius of gyration

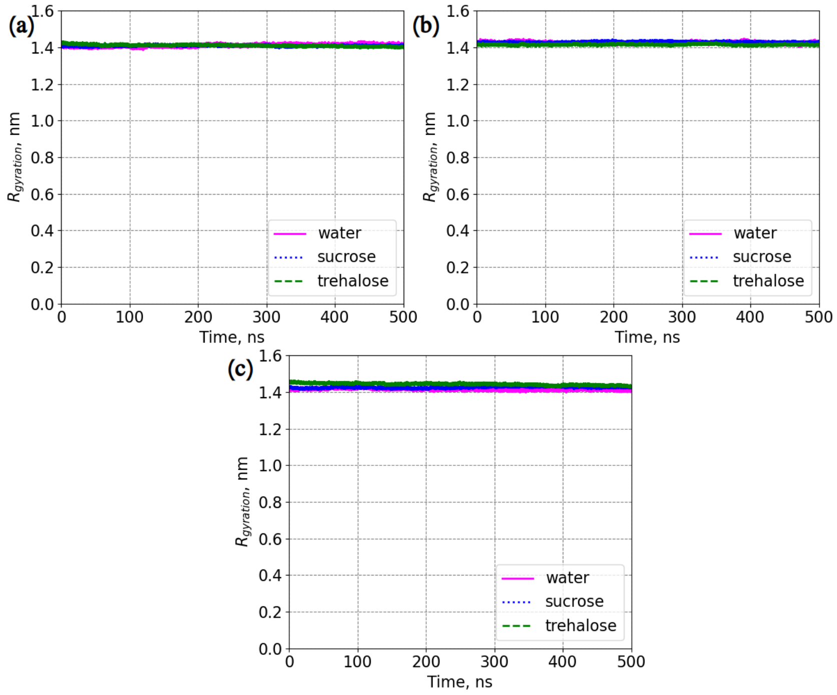

Figure S4 Average radius of gyration for proteins. (a) Systems: LYS., LYS.+SUC., LYS.+TRE. (b) Systems: LYS.+NaCl, LYS.+SUC.+NaCl, LYS.+TRE.+NaCl. (c) Systems: LYS.+$ZnCl_2$, LYS.+SUC.+$ZnCl_2$, LYS.+TRE.+$ZnCl_2$. "Water" stands for systems without disaccharides. RMSD was computed for each protein and then averaged for 4 molecules.

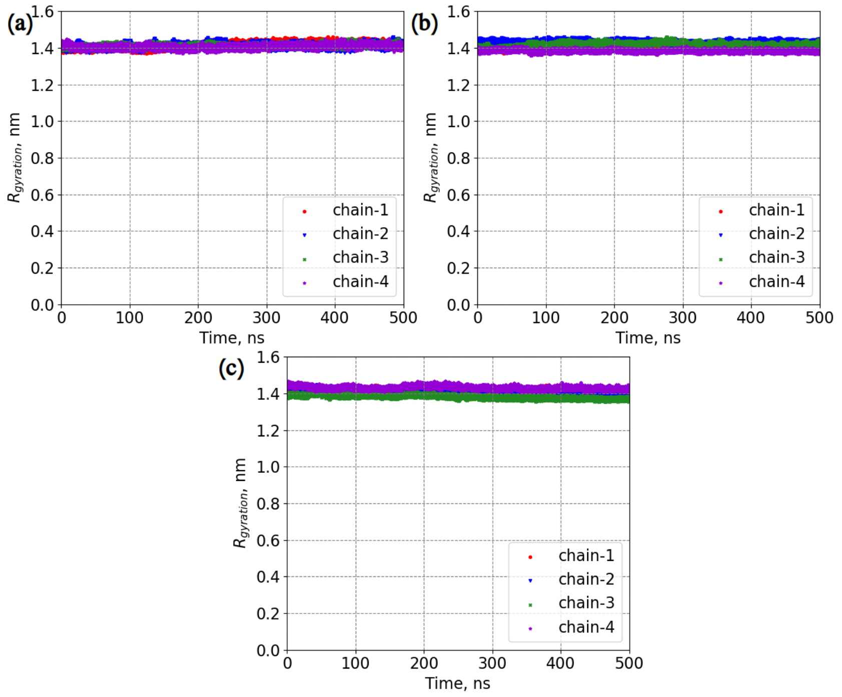

Figure S5 Radius of gyration for protein chains. (a) Systems: LYS. (b) LYS.+SUC. (c) LYS.+TRE. Radius of gyration was computed for each protein.

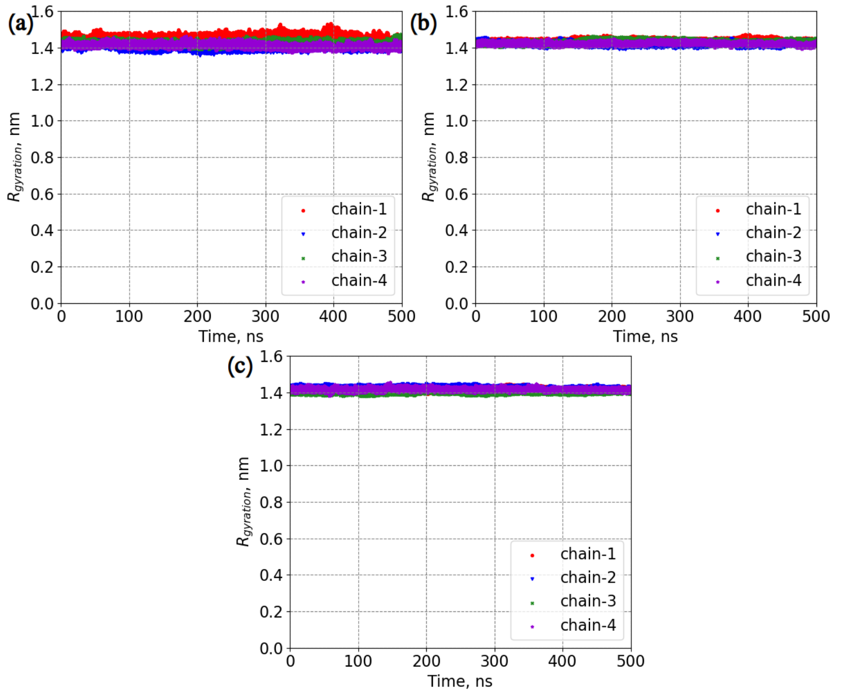

Figure S6 Radius of gyration for protein chains. (a) Systems: LYS.+NaCl. (b) LYS.+SUC.+NaCl. (c) LYS.+TRE.+NaCl. adius of gyration was computed for each protein.

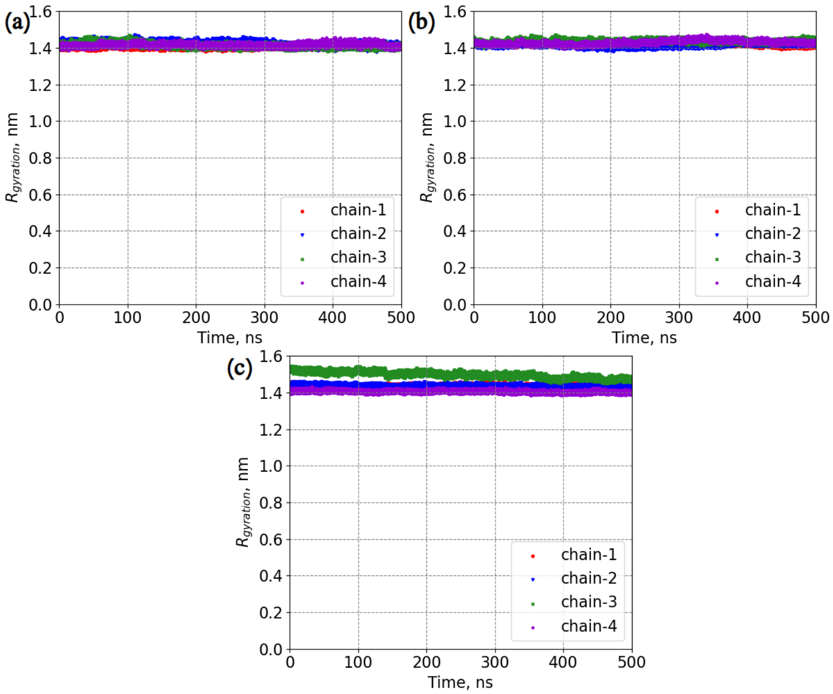

Figure S7 adius of gyration for protein chains. (a) Systems: LYS.+$ZnCl_2$. (b) LYS.+SUC.+$ZnCl_2$. (c) LYS.+TRE.+$ZnCl_2$. adius of gyration was computed for each protein.

3 Self-intermediate scattering functions

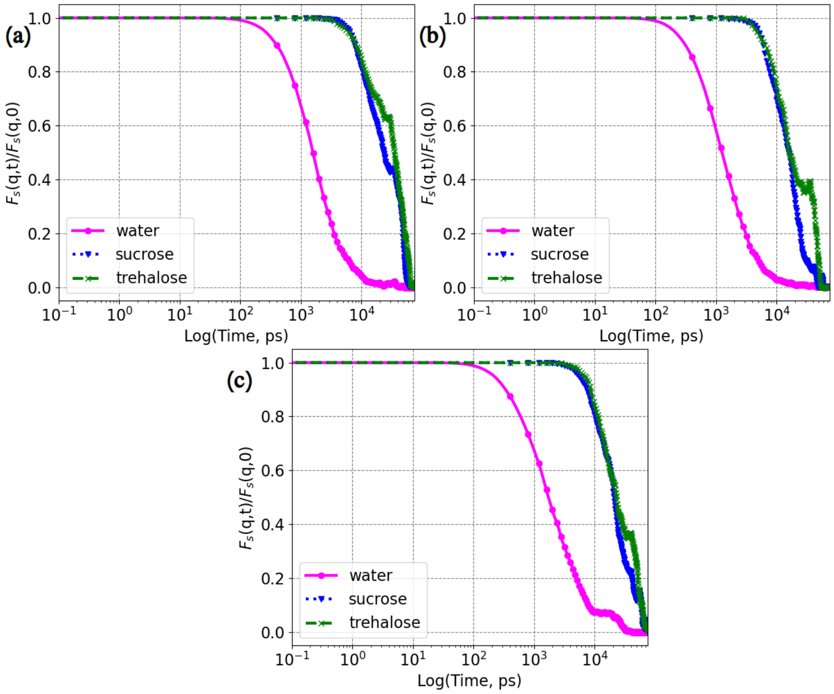

Figure S8 Self-intermediate scattering function for the protein's back-bone for q=15.71 nm^{-1}. (a) Systems: LYS., LYS.+SUC., LYS.+TRE. (b) Systems: LYS.+NaCl, LYS.+SUC.+NaCl, LYS.+TRE.+NaCl. (c) Systems: LYS.+$ZnCl_2$, LYS.+SUC.+$ZnCl_2$, LYS.+TRE.+$ZnCl_2$. "Water" stands for systems without disaccharides.

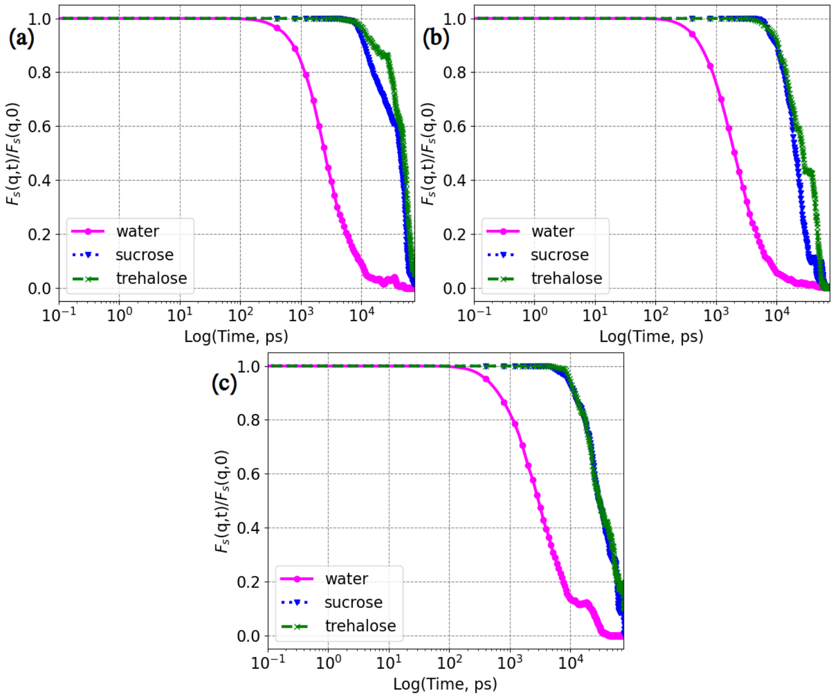

Figure S9 Self-intermediate scattering function for the protein's back-bone for q=12.57 nm^{-1}. (a) Systems: LYS., LYS.+SUC., LYS.+TRE. (b) Systems: LYS.+NaCl, LYS.+SUC.+NaCl, LYS.+TRE.+NaCl. (c) Systems: LYS.+$ZnCl_2$, LYS.+SUC.+$ZnCl_2$, LYS.+TRE.+$ZnCl_2$. "Water" stands for systems without disaccharides.

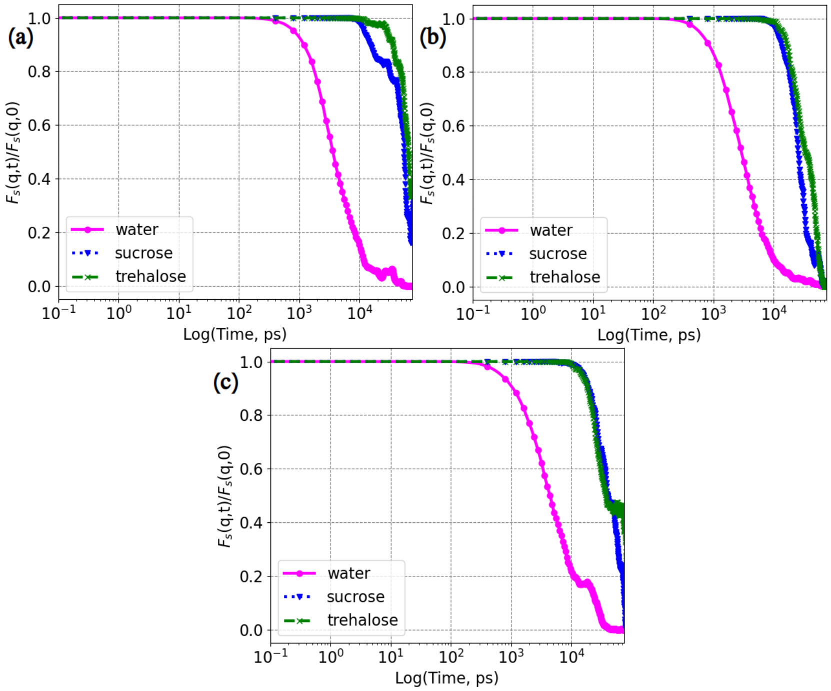

Figure S10 Self-intermediate scattering function for the protein's back-bone for q=10.47 nm^{-1}. (a) Systems: LYS., LYS.+SUC., LYS.+TRE. (b) Systems: LYS.+NaCl, LYS.+SUC.+NaCl, LYS.+TRE.+NaCl. (c) Systems: LYS.+$ZnCl_2$, LYS.+SUC.+$ZnCl_2$, LYS.+TRE.+$ZnCl_2$. "Water" stands for systems without disaccharides.

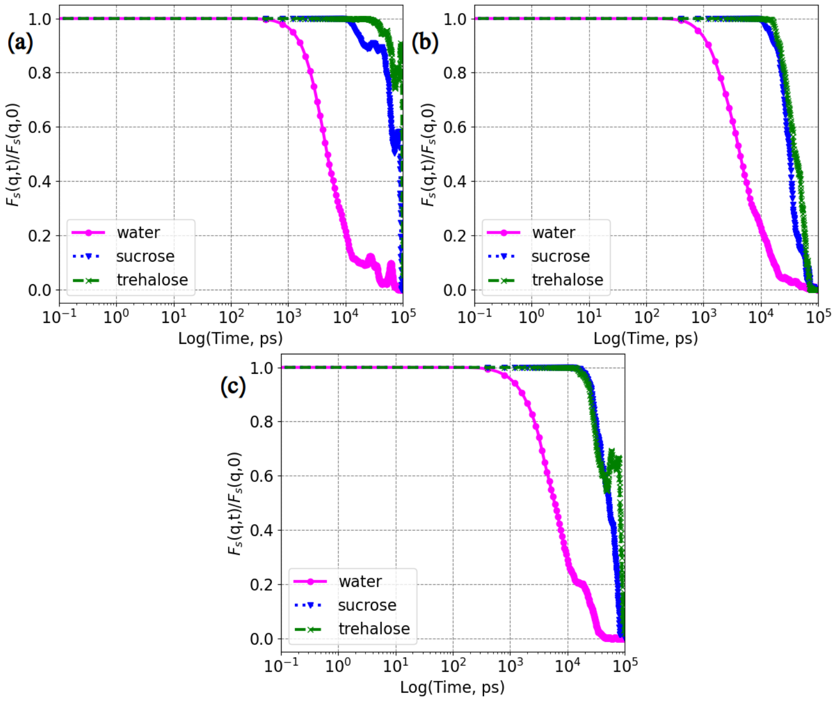

Figure S11 Self-intermediate scattering function for the protein's back-bone for q=8.98 nm^{-1}. (a) Systems: LYS., LYS.+SUC., LYS.+TRE. (b) Systems: LYS.+NaCl, LYS.+SUC.+NaCl, LYS.+TRE.+NaCl. (c) Systems: LYS.+$ZnCl_2$, LYS.+SUC.+$ZnCl_2$, LYS.+TRE.+$ZnCl_2$. "Water" stands for systems without disaccharides.

4 Average number of hydrogen bonds per 1 water molecule

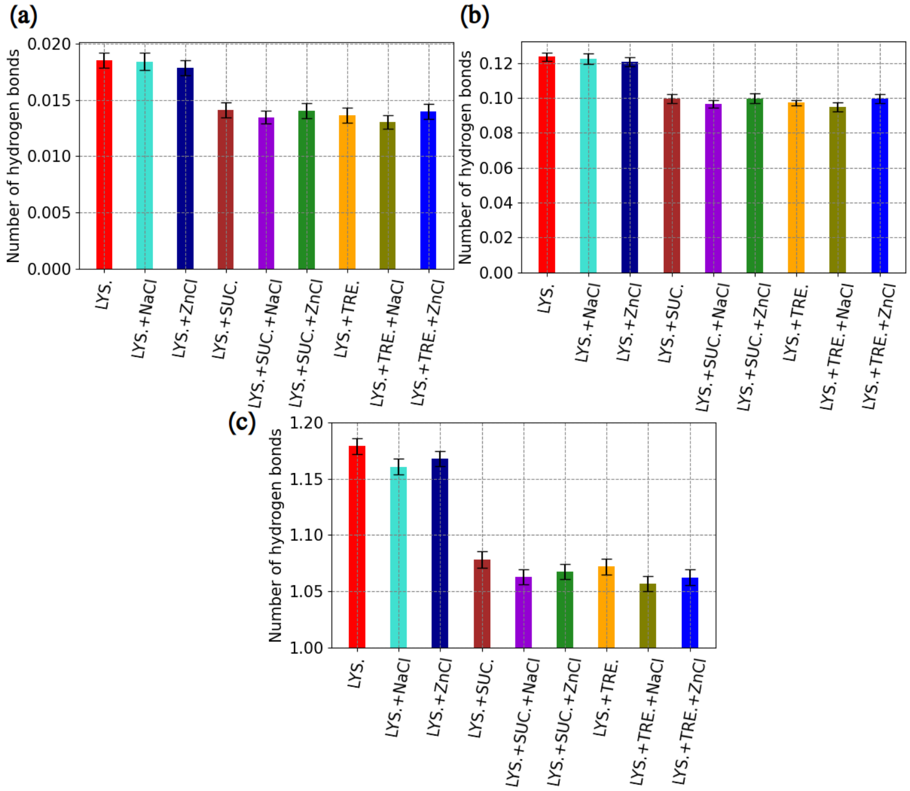

Figure S12 Average number of hydrogen bonds per 1 water molecule. (a) Protein-water-protein (here values are per protein molecule): bridging intra-protein bonds with water. (b) Protein-water. (c) Water-water.
